# Female Gender and Differences in Outcome after Isolated Coronary Artery Bypass Graft Surgery: Does Age Play a Role?

**DOI:** 10.1371/journal.pone.0145371

**Published:** 2016-02-04

**Authors:** Rawa Arif, Mina Farag, Victor Gertner, Gabor Szabó, Alexander Weymann, Gabor Veres, Arjang Ruhparwar, Raffi Bekeredjian, Tom Bruckner, Matthias Karck, Klaus Kallenbach, Carsten J. Beller

**Affiliations:** 1 Department of Cardiac Surgery, Heart Center Heidelberg, University Hospital Heidelberg, Heidelberg, Germany; 2 Department of Cardiology, Angiology and Pneumology, Heart Center Heidelberg, University Hospital Heidelberg, Heidelberg, Germany; 3 Institute of Medical Biometry and Informatics, Heart Center Heidelberg, University of Heidelberg, Heidelberg, Germany; University of Crete, GREECE

## Abstract

**Introduction:**

Female gender is a known risk factor for early and late mortality after coronary artery bypass graft surgery (CABG). Higher age of women at operation may influence outcome, since age per se is also an important risk factor. The purpose of our study was to analyze possible gender differences in outcome after isolated CABG in different age groups to delineate the impact of female gender and age.

**Methods:**

All patients over 60 years of age undergoing isolated CABG at our department during 2001 and 2011 were included and categorized by age into sexagenarians (2266, 16.6% women), septuagenarians (2332, 25.4% women) and octogenarians (374, 32% women) and assessed by gender for 30-day and 180-day mortality.

**Results:**

Thirty-day mortality was significantly higher in women only amongst septuagenarians (7.1 vs. 4.7%, p = 0.033). Same differences apply for 180-day mortality (12.3 vs. 8.2%, p = 0.033) and estimated one-year survival (81.6 ± 4.2 vs. 86.9 ± 2.2%, p = 0.001). Predictive factors for 30-day mortality of septuagenarian were logistic EuroSCORE (ES) (p = 0.003), perioperative myocardial infarction (MI) (p<0.001), pneumonia (p<0.001), abnormal LV-function (p<0.04) and use of LIMA graft (p<0.001), but not female gender. However, female gender was found to be an independent predictor for 180-day mortality (HR 1.632, p = 0.001) in addition to ES, use of LIMA graft, perioperative MI, pneumonia and abnormal LV function (HR 1.013, p = 0.004; HR 0.523, p<0.001; HR 2.710, p<0.001; HR 3.238, p<0.001; HR 2.013, p<0.001).

**Conclusion:**

Women have a higher observed probability of early death after CABG in septuagenarians. However, female gender was not found to be an independent risk factor for 30-day, but for 180-day survival. Therefore, reduction of high impact risk factors such as perioperative MI and enhancement of LIMA use should be future goals. In view of our findings, decision for surgical revascularization should not be based on gender.

## Introduction

In the past, female gender was reported to be an independent predictor for early [1–5] and late mortality after coronary artery bypass graft surgery (CABG) [6, 7]. In-hospital mortality after CABG was up to twice as high in women compared to men [8]. Older age, smaller body size and coronary artery diameters, as well as higher incidence of comorbidities such as diabetes, arterial hypertension and hypercholesterolemia were found to be responsible for these gender-specific differences in outcome after CABG [8]. These gender differences in late mortality were more pronounced, when only patients less than 70 years of age were analyzed [7]. Thus, the most common risk model for cardiosurgical procedures in Europe, the EuroSCORE, as well as enhanced models such as the German CABG Score imbedded female gender as a key variable for adverse outcome [9, 10].

However, there are inconsistent reports in current literature. In very high-risk patients, female and male mortality rates after CABG were found to be similar, whereas female gender was otherwise an independent predictor of higher operative CABG mortality [5]. In other studies, no gender-specific differences for perioperative mortality [11] and for long-term survival after CABG were observed [1, 12–14]. Older age, previous CABG, previous MI and diabetes were independent risk factors for higher mortality, but female gender was not [15]. Similarly, a recent study found that in septuagenarians and octogenarians female gender was not associated with increased risks for morbidity and 30-day mortality after isolated CABG, isolated valve replacement or combined procedures [16]. Gender differences for mortality after CABG were less pronounced with increasing patients’ age [17]. Some studies even reported better long-term survival for women after CABG [18, 19].

These confounding findings prompted the present retrospective study. The aim was to analyze possible gender differences in 30-day and 180-day mortality after isolated CABG in different age groups: sexagenarians, septuagenarians and octogenarians.

## Patients and Methods

### Study Design

An analysis of our center’s contemporary register (between 2001 and 2011) included 4972 consecutive patients aging 60 years and above, who underwent isolated, primary CABG. Data collection was done by chart review. Follow-up was obtained after written consent and approval of institutional review board Ethikkommission der Universität Heidelberg (Ethics committee University of Heidelberg) (S-286/2010) through contact with the local population administration office, family doctor or the patient/family directly (only adult patients who are legally competent were included). In accordance with the local ethic committee, the requirement of individual patient consent was waived because of the study’s retrospective design and the data collection from routine care. All data were anonymized and de-identified prior to analysis. Follow-up was complete 95.2% after 30 days and 92.3% after 180 days with an mean time of 202.29 ± 224 days.

### Patients’ demographics

Patients were diagnosed with single-, double- or triple-vessel coronary heart disease. The prevalence of left main coronary artery stenosis and triple-vessel disease was analyzed. Patients with concomitant cardiac or aortic procedures were not included in our study. Ejection fraction was examined by referring physicians using standard echocardiographic measurements or during angiography. Abnormal LV function was assessed by echocardiographic findings including e.g. diastolic dysfunction independent from ejection fraction. Obesity was defined, if BMI reached 30 kg/m^2^ or more. Previous myocardial infarction is defined as a clinical history of ST elevation and non-ST elevation myocardial infarction. Psychiatric disorders are defined as a clinical history of disorders listed in ICD 10 Chapter V. Demographic data are listed in Table 1.

**Table 1:** Preoperative baseline characteristics.

	sexagenarian (n = 2266)			septuagenarian (n = 2332)			octogenarian (n = 374)		
Variable n (%) or mean ± SDM	Women (n = 377)	Men (n = 1889)	*P*	Women (n = 592)	Men (n = 1740)	*P*	Women (n = 121)	Men (n = 253)	*P*
Unstable angina	113 (30)	487 (26)	.11	178 (30)	440 (25)	.024	44 (36)	58 (23)	.009
Triple-vessel disease	219 (58)	1318 (70)	.000	412 (70)	1299 (75)	.013	96 (80)	211 (83)	.387
LMS stenosis >50%	180 (50)	1019 (55)	.073	309 (53)	952 (56)	.210	74 (63)	161 (65)	.727
Abnormal LV function	131 (35)	901 (48)	.000	264 (45)	908 (52)	.001	57 (47)	140 (55)	.151
Ejection fraction			.000			.003			.322
Good (>50%)	245 (65)	983 (52)		326 (55)	826 (48)		64 (53)	113 (45)	
Moderate (30–49%)	102 (27)	657 (35)		195 (33)	638 (37)		40 (33)	96 (38)	
Poor (<30%)	29 (7.7)	244 (13)		69 (11.7)	270 (16)		17 (14)	44 (17)	
Previous cardiac surgery	8 (2.1)	54 (2.9)	.493	6 (1)	48 (2.8)	.016	0 (0)	1 (1.6)	.309
**Comorbidities**									
Previous myocardial infarction	166 (44)	955 (51)	.021	317 (54)	932 (54)	1.0	72 (60)	141 (56)	.502
Atrial fibrillation			.444			.235			.216
Intermittent	17 (4.5)	94 (5)	.130	55 (9.3)	165 (9.5)	.735	14 (11.6)	23 (9.1)	.324
Persistent	4 (1.1)	22 (1.2)	.070	12 (2)	49 (2.8)	.684	3 (2.5)	9 (3.6)	.633
Chronic	3 (0.8)	37 (2)	.568	12 (2)	60 (3.4)	.941	3 (2.5)	19 (7.5)	.193
Preoperative dialysis	10 (2.7)	29 (1.5)	.130	13 (2.2)	34 (2)	.735	1 (0.8)	0 (0)	.324
s/p PTCA	105 (28)	617 (33)	.070	195 (33)	557 (32)	.684	23 (19)	64 (25)	.633
History of stroke	40 (10.6)	181 (9.6)	.568	69 (11.7)	207 (12)	.941	14 (11.6)	40 (16)	.193
Peripheral vascular disease	59 (16)	344 (18)	.239	76 (13)	333 (19)	.001	24 (20)	39 (15)	.303
Diabetes	146 (39)	662 (35)	.175	270 (46)	659 (38)	.001	53 (44)	74 (29)	.007
Hypertension	357 (89)	1754(86)	.262	565 (96)	1638 (94)	.171	118 (98)	243 (96)	.560
Dyslipidemia	336 (95)	1611(93)	.061	501 (85)	1460 (84)	.693	100 (83)	200 (79)	.402
History of psychiatric disorders	17 (4.5)	43 (2.3)	.022	27 (4.6)	33 (1.9)	.001	3 (2.5)	6 (2.4)	1.00
Smoking	119 (32)	1099(59)	.000	94 (16.2)	830 (49)	.000	12 (10.2)	101 (40)	.000
Obesity	224 (59)	855 (45)	.000	324 (55)	699 (40)	.000	45 (38)	68 (27)	.040
COPD	62 (16)	375 (10)	.117	97 (17)	378 (22)	.005	16 (13)	54 (21)	.087
Preoperative creatinine level	0.96±0.78	1.1±0.75	.000	1.08±0.85	1.19±0.77	.004	1.07±0.56	1.20±0.42	.019
EuroSCORE log. mean	9.46±7.5	7.9±7.9	.001	17.8±13.7	14.2±12.3	.000	25.50±17.5	21.2±16.3	.018

*LMS* left main stenosis; *LV* left ventricular; *PTCA* percutaneous transluminal coronary angioplasty; *COPD* chronic obstructive pulmonary disease

### Operative Protocol

All procedures were performed via full median sternotomy. Indication for operation were elective, urgent, emergent (immediate operation after admission) or ultima ratio. Operations were performed with the aid of standard extracorporeal circulation (ECC) employing single cannulation of the ascending aorta and right atrium. Activated clotting time (ACT) was set 400 seconds by intraoperative heparinization before cannulation. A membrane oxygenator was applied and surgery was performed at different levels of hypothermia. Operation data are displayed in Table 2. Erythrocyte concentrate, fresh frozen plasma and platelet transfusions were administered, if required. Proximal anastomoses were mainly performed under partial aortic clamping. Total arterial revascularization, which include single LIMA on left anterior descending (LAD) bypass and multiple bypass grafts using left and/or right IMA and/or use of radial artery were analyzed and compared to conventional revascularization within study groups (Table 2). The patients were later transferred to the intensive care unit (ICU) and received standard hemodynamic monitoring and mechanical ventilation after surgery. Anti-platelet agents were started at the first postoperative day. Earlier administration of anti-platelet agents followed specific requirements by the operating surgeon due to extended anastomose or endarterectomy. Several postoperative variables were documented and included in statistical analysis (Table 3). Perioperative myocardial infarction was defined as elevated myocardial enzymes including creatinine kinase (CK), creatinine kinase MB (CK-MB) and troponin T accompanied by significant ischemic changes of electrocardiogram (ECG) and/or echocardiographic evidence of consecutive loss of viable myocardium and/or significant haemodynamic deterioration and/or angiographically documented new graft or native coronary occlusion. However, definite thresholds of cardiac enzymes are not given, since diagnosis was made on individual clinical decision. Respiratory insufficiency indicates need of forced respiratory therapy due to reduced Horowitz index. Psychoneurotic complications indicate postoperative delirium. Gastrointestinal event indicates sub ileus, mechanical or paralytic ileus, pancreatitis, cholecystitis, cholangitis, gastrointestinal bleeding and mesenteric ischemia, which may have led to laparotomy in some cases. Infection indicates general infection including wound infection, bacteremia, mediastinits, systemic fungal infection, peritonitis, catheter or urine tract infection etc.

**Table 2:** Intraoperative risk factors for patients undergoing bypass surgery.

	sexagenarian (n = 2266)			septuagenarian (n = 2332)			octogenarian (n = 374)		
Variable n (%) or mean ± SDM	Women (n = 377)	Men (n = 1889)	*P*	Women (n = 592)	Men (n = 1740)	*P*	Women (n = 121)	Men (n = 253)	*P*
Indication			.367			.234			.714
Elective	49 (13)	218(11.5)		47 (7.9)	160 (9.2)		11(9.1)	19 (7.5)	
Urgent	277 (74)	1373(73)		422 (71)	1281 (74)		82 (68)	185(73)	
Emergent	49 (13)	294 (16)		116 (20)	282 (16)		26 (22)	44 (17)	
Ultima ratio	2 (0.5)	4 (0.2)		7 (1.2)	17 (1)		2 (1.7)	5 (2)	
Use of LIMA graft	341 (91)	1693 (90)	.710	478 (81)	1505 (87)	.001	69 (57)	178(70)	
Total arterial revascularization	33 (8.8)	92 (4.9)	.004	28 (4.7)	60 (3.4%)	.170	5 (4.1)	10 (4)	1.0
Cardiopulmonary bypass, min.	100.4±43.5	98.3±37.4	.339	98.9±41.7	99.6±38.1	.701	95.7±42	93±32.8	.531
Aortic cross-clamp, min.	54.1±21.5	53.5±20.7	.64	51.1±20.0	53.7±20.7	.007	47±17.7	49±18.7	.409
No. of distal anastomoses	2.3±0.7	2.4±0.6	.085	2.3±0.6	2.35±0.6	.531	2.2±0.7	2.2+0.6	.987

*LIMA* left internal mammary artery

**Table 3:** Postoperative data and complications.

	sexagenarian (n = 2266)			septuagenarian (n = 2332)			octogenarian (n = 374)		
Variable n (%) or mean ± SDM	Women (n = 377)	Men (n = 1889)	*P*	Women (n = 592)	Men (n = 1740)	*P*	Women (n = 121)	Men (n = 253)	*P*
Perioperative MI	32 (8.5)	97 (5.1)	.014	45 (7.6)	76 (4.4)	.004	4 (3.3)	12 (4.7)	.597
Reoperation for bleeding	24 (6.4)	85 (4.5)	.146	28 (4.7)	102 (5.9)	.351	6 (4.6)	21 (8.3)	.290
Respiratory Insufficiency	41 (10.9)	154 (8.2)	.088	106 (17)	247 (14)	.083	19 (16)	39 (15)	1.000
IABP perioperative	33 (8.8)	134 (7.1)	.280	68 (11.5)	156 (9)	.076	17 (14)	21 (8.3)	.100
Stroke	2 (0.5)	11 (0.6)	1.000	2 (0.3)	16 (0.9)	.274	0 (0)	4 (1.6)	.309
Postoperative VT	36 (9.5)	183 (9.7)	1.000	71 (12)	202 (11.6)	.824	16 (13)	27 (10.7)	.491
Psychoneurotic complication	48 (12.7)	270 (14)	.465	92 (16)	385 (22)	.000	19 (16)	66 (26)	.025
Reoperation for tamponade	22 (5.8)	80 (4.2)	.174	21 (3.6)	91 (5.2)	.119	4 (3.3)	18 (7.1)	.165
Instable sternum	2 (0.5)	22 (1.2)	.409	5 (0.8)	23 (1.3)	.512	0 (0)	4 (1.6)	.309
Cerebrovascular accident	13 (3.4)	61 (3.2)	.874	25 (4.2)	60 (3.4)	.376	3 (2.5)	14 (5.5)	.288
Gastrointestinal event	16 (4.2)	80 (4.2)	1.000	32 (5.4)	119 (7.4)	.110	6 (5)	23 (9.1)	.215
Reanimation	7 (1.9)	47 (2.5)	.580	11 (1.9)	43 (2.5)	.434	2 (1.7)	8 (3.2)	.510
Wound revision	7 (1.9)	16 (0.8)	.088	14 (2.4)	24 (1.4)	.130	3 (2.5)	4 (1.6)	.686
Laparatomy	2 (0.5)	23 (1.2)	.414	13 (2.2)	30 (1.7)	.480	2 (1.7)	5 (2)	1.000
Infection	44 (11.7)	195 (10.3)	.462	86 (15)	304 (18)	.111	20 (17)	49 (19)	.570
Mediastinitis	0 (0)	5 (0.3)	1.000	2 (0.3)	3 (0.2)	.606	0 (0)	2 (0.8)	1.000
Sepsis	7 (1.9)	48 (2.5)	.582	28 (4.7)	74 (4.3)	.642	7 (5.8)	12 (4.7)	.626
Pneumonia	30 (8)	120 (6.5)	.310	39 (6.6)	186(10.7)	.003	15 (12.4)	39 (15)	.530
Wound infection (thorax)	2 (0.5)	12 (0.6)	1.000	8 (1.4)	16 (0.9)	.353	0 (0)	2 (0.8)	1.000
Venous catheter infection	5 (1.3)	16 (0.8)	.376	7 (1.2)	28 (1.6)	.560	1 (0.8)	2 (0.8)	1.000
Urinary tract infection	8 (2.1)	13 (0.7)	.015	16 (2.7)	41 (2.4)	.645	5 (4.1)	3 (1.2)	.118
Systemic fungal infection	3 (0.8)	5 (0.3)	.134	5 (0.8)	12 (0.7)	.780	0 (0)	2 (0.8)	1.000
Other infection	4 (1.1)	39 (2.1)	.298	22 (3.7)	68 (3.9)	.902	2 (1.7)	9 (3.6)	.514
Mean Intubation, h	37± 91	33 ± 128	.593	53 ± 164	44 ± 139	.227	45± 97	59 ± 148	.342

*MI* myocardial infarction; *IABP* intra aortic balloon pump; *VT* ventricular tachycardia

### Statistical analysis

Patients were categorized by age into sexagenarians, septuagenarians and octogenarians and assessed by gender for differences in baseline characteristics, operative and perioperative data, 30-day and 180-day mortality and estimated long-term survival. Descriptive statistics were used for patient characteristics and to compare variables. Categorical data were reported as frequency distributions and percentages. Continuous data were presented as mean ± standard deviation. Unpaired Student’s t test was used for comparison of continuous variables. Fisher’s exact test or Chi square test were used for comparison of categorical variables. Univariate logistic regression was performed to detect possible risk factors for 30-day and 180-day mortality in septuagenarians. Significant factors were further included and analyzed by the Cox proportional hazards model to determine perioperative risk factors regarding 30-day and 180-mortality. Multivariate logistic regression analysis was used as a sensitivity test to confirm the results of the Cox regression model. Survival function was estimated by use of Kaplan-Meier curve. A two-tailed p value less than 0.05 was considered significant. SPSS 22.0 software (SPSS, Inc, Chicago, Ill.) was used for all statistical analysis.

## Results

4972 patients were enrolled (21.92% women). Patients were divided according to age and gender into sexagenarians (female 377, male 1889), septuagenarians (female 592, male 1740) and octogenarians (female 121, male 253). Baseline characteristics are shown in Table 1. No significant differences were found between genders in baseline prevalence of left main stenosis or atrial fibrillation. Previous myocardial infarction was significantly more frequent amongst sexagenarian men (50.6% vs. 44%; p = 0.021) alongside COPD (21.8% vs. 16.5%; p = 0.005). Left ventricular ejection fractions showed significant differences in sexa- and septuagenarians indicating women to present with relatively better LV function.

Mean logistic EuroSCORE was significantly higher in women of all age groups, as expected due to the high impact of gender within the scoring system. Diabetes mellitus was significantly higher in women compared to men only in septua- and octogenarians (46% vs. 38%; p = 0.001 and 44% vs. 29%; p = 0.007) as it was among sexagenarians, but without reaching statistical significance. The prevalence of smoking was higher in men of all age groups. Renal dysfunction with need of dialysis was equally found in all groups without statistical difference. Preoperative mean creatinine levels were significantly higher in women of all age groups (p<0.001 in sexa-; p = 0.004 in septua-; p = 0.019 in octogenarians).

Operative data are listed in Table 2. Urgency of operation did not differ significantly among the study groups (sexagenarians p = 0.367, septuagenarians p = 0.234, octogenarians p = 0.714). Cross-clamp time was longer in septuagenarian men (54 min vs. 51 min; p = 0.004). One of the major differences is that the LIMA bypass graft was more often used in septua- and octogenarian men compared to women (86.5% vs. 80.7%, p = 0.001; 70% vs. 57%, p = 0.014), whereas the mean number of distal anastomoses did not differ between the study groups (sexagenarians p = 0.085, septuagenarians p = 0.531, octogenarians p = 0.987). Further investigation revealed that the procedure of total arterial revascularization was followed without significant difference in septua- and octogenerians (p = 0.170 and p = 1.0). The amount of performed total arterial revascularization was higher in sexagenarian women compared to men (8.8% vs. 4.9%; p = 0.004).

Postoperative variables and complications are presented in Table 3. No significant differences were found during the operative course among sexagenarians, except for urine tract infection, which occurred more in women as expected (2.1% vs. 0.7%, p = 0.015). Neither cerebrovascular incidence (sexagenarians p = 0.874, septuagenarians p = 0.376, octogenarians p = 0.288) nor stroke (sexagenarians p = 1, septuagenarians p = 0.274, octogenarians p = 0.309) differed among age groups. Interestingly, the incidence of infections, sepsis, mediastinitis and especially thoracic wound infections (sexagenarians p = 1, septuagenarians p = 0.353, octogenarians p = 1) did also not differ between women and men among all age groups. However, a major difference is the occurrence of perioperative myocardial infarction. Significantly more sexa- and septuagenarian women suffered from perioperative MI compared to men (8.5% vs. 5.1%, p = 0.014; 7.6% vs. 4.4%, p = 0.004). Furthermore, septuagenarian men developed pneumonia more often compared to women (10.7% vs. 6.6%, p = 0.003). However, mean intubation time was relatively comparable in that age group (53 min. vs. 44 min., p = 0.227). Sexagenarians and octogenarians showed statistically likely incidence of postoperative pneumonia (sexagenarians p = 0.310, octogenarians p = 0.530) and ventilation time (sexagenarians p = 0.593, octogenarians p = 0.342).

### Outcome

**Fig 1:**
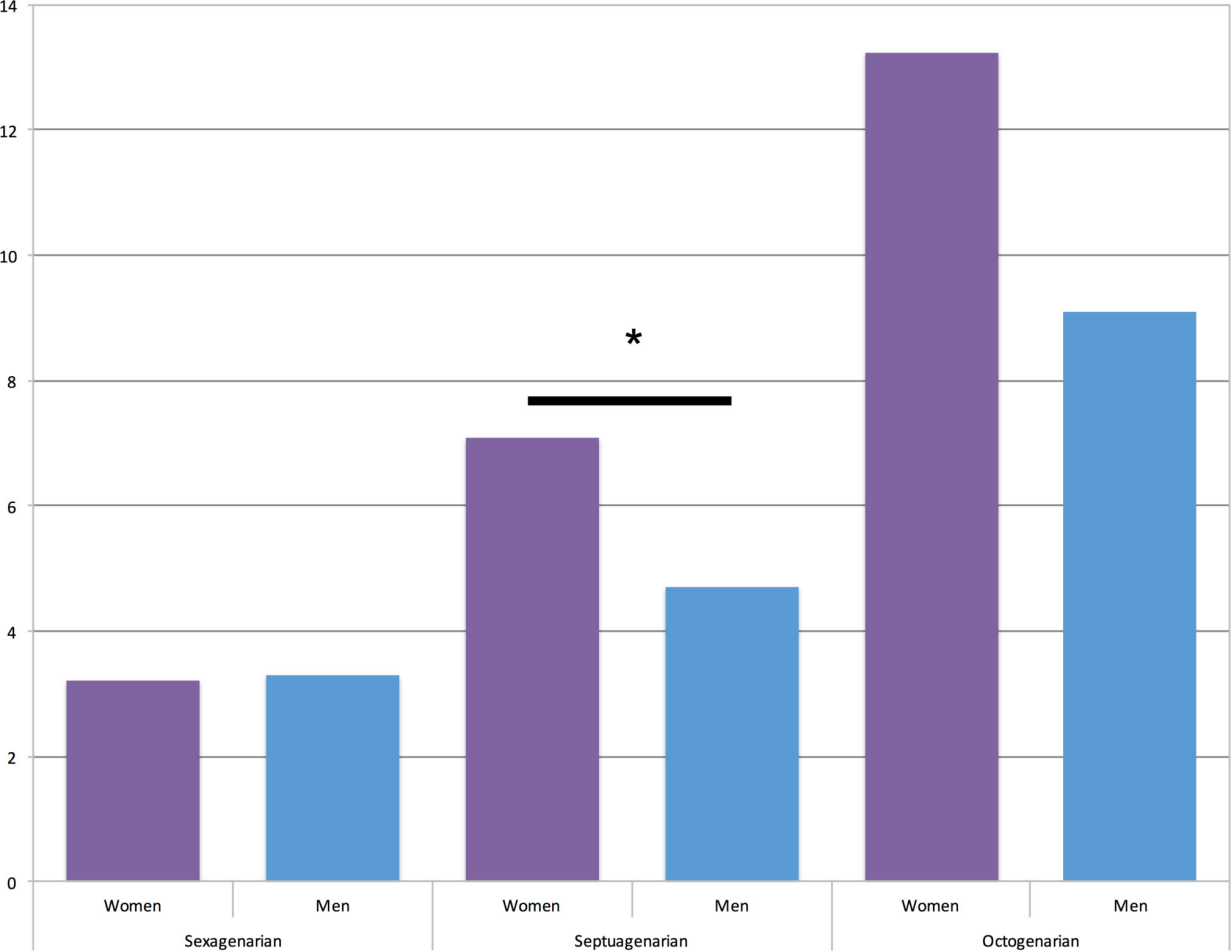
30-day mortality of study cohort: sexagenarians (3.2% vs. 3.3%, p = 1.0); septuagenarians (7.1% vs. 4.7%, *p = 0.003) and octogenarian (12.2% vs. 9.1%, p = 0.277).

**Fig 2:**
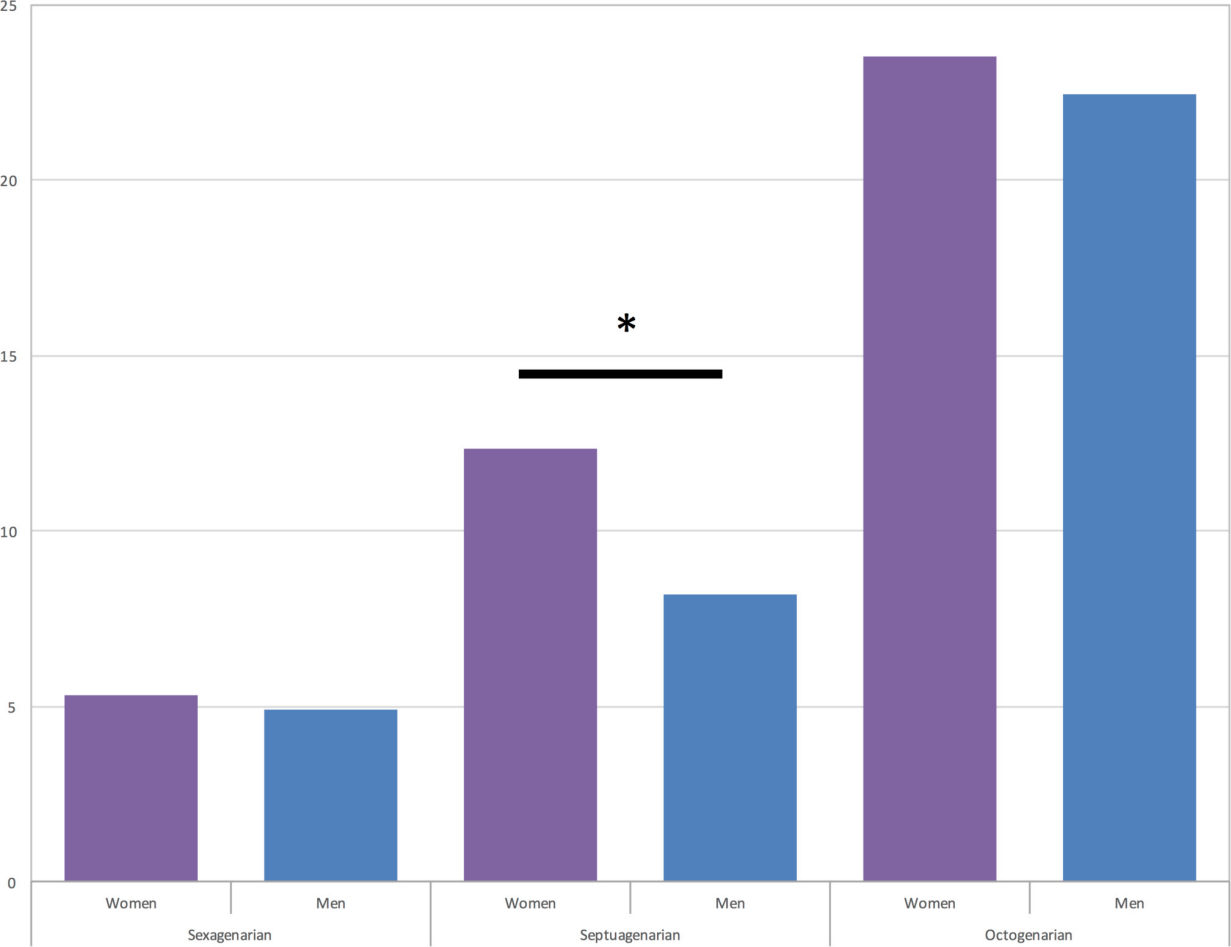
180-day mortality of study cohort: sexagenarians (4.3% vs. 4.9%, p = 0.696); septuagenarians (12.3% vs. 8.2%, *p = 0.003) and octogenarian (23.5% vs. 22.4%, p = 0.761).

30-day and 180-day mortality of our subgroups are depicted in Figs 1 and 2. 30-day Mortality was significantly higher in women among septuagenarians (7.1% vs. 4.7%, p = 0.033), as well as 180-day mortality (12.3% vs. 8.2%, p = 0.033). In octogenarians relative values of 30-day mortality showed the same tendencies without reaching statistical significance (13.2% vs. 9.1%, p = 0.277), whereas 180-day mortality tended to similar outcomes of women and men (23.5% vs. 22.4%, p = 0.761). Among sexagenarians 30-day mortality was almost equal (3.2% vs. 3.3%, p = 1.0), as well as 180-day mortality was (5.3% vs. 4.9%, p = 0.696).

Since descriptive analysis revealed significant differences in short- and mid-term outcome only among septuagenarians, this subgroup was further investigated by Cox proportional hazard model after proving relevance of included variables by univariate logistic regression analysis.

On univariate analysis (Table 4) female gender, logistic EuroSCORE, peripheral artery disease (PAD), abnormal left ventricular (LV) function, postoperative pneumonia, perioperative myocardial infarction (MI), previous heart surgery and the non-use of the left internal thoracic artery (LIMA) as a bypass graft were significant predictors of 30-day mortality in septuagenarians. However, the incidence of perioperative morbidity in terms of neurological and gastrointestinal complications, were not significant.

**Table 4:** Univariate analyses of variables influencing 30-day mortality of septuagenarians.

Potential Risk Factor	Univariate		
	Odds ratio (OR)	95% Confidence interval (CI)	*p* value
Women	1.544	(1.051–2.262)	.027
Abnormal LV function	2.292	(1.550–3.391)	.000
PAD	2.100	(1.399–3.152)	.000
Use of LIMA graft	.270	(.184–.396)	.000
Pneumonia	3.989	(2.617–6.081)	.000
Perioperative MI	5.223	(3.200–8.524)	.000
Previous HS	3.241	(1.495–7.026)	.003
EuroScore logistic	1.043	(1.033–1.053)	.000

*LV* left ventricular; *LIMA* left internal mammary artery; *MI* myocardal infarction; *PAD* peripheral artery disease

Cox proportional hazard model was used to identify risk-factors for 30-day and 180-day mortality among septuagenarians (Table 5). Abnormal LV-function, non-use of the LIMA graft, pneumonia, perioperative myocardial infarction and logistic EuroScore remained significant predictive risk factors for 30-day mortality. However, female gender (p = 0.084) did not remain a risk factor for 30-day mortality alongside PAD and previous heart surgery. The results were mainly confirmed by multivariate logistic regression (Table 6). For 180-day mortality the same results were detected, but, furthermore, surprisingly female gender became a significant risk factor with an HR of 1.632 (CI 1.212–2.200, p = 0.001). These results could again be confirmed by multivariate logistic regression (Table 6).

**Table 5:** Cox logistic regression analyses of variables influencing 30-day mortality and 180-day mortality of septuagenarians.

Potential Risk Factor	30days			180days		
	Hazard Ratio (HR)	95% Confidence interval (CI)	*p* value	Hazard Ratio (HR)	95% CI	*p* value
Women	1.421	(0.954–2.117)	.084	**1.632**	**(1.212–2.200)**	**.001**
**Abnormal LV function**	**1.566**	**(1.022–2.400)**	**.040**	**2.013**	**(1.448–2.798)**	**.000**
PAD	1.482	(0.970–2.264)	.069	1.359	(0.978–1.890)	.068
**Use of LIMA graft**	**0.480**	**(0.321–0.717)**	**.000**	**0.529**	**(0.389–0.720)**	**.000**
**Pneumonia**	**2.284**	**(1.488–3.507)**	**.000**	**3.238**	**(2.364–4.434)**	**.000**
**Perioperative MI**	**3.367**	**(2.090–5.425)**	**.000**	**2.710**	**(1.834–4.003)**	**.000**
Previous HS	1.869	(0.891–3.920)	.098	1.113	(0.561–2.209)	.759
**EuroScore logistic**	**1.017**	**(1.006–1.028)**	**.003**	**1.013**	**(1.004–1.022)**	**.004**

*LV* left ventricular; *LIMA* left internal mammary artery; *MI* myocardial infarction; *PAD* peripheral artery disease; *HS* heart surgery

**Table 6:** Multivariate regression analyses of variables influencing 30-day mortality and 180-day mortality of septuagenarians.

Potential Risk Factor	30days			180days		
	Odds Ratio (OR)	95% Confidence interval (CI)	*p* value	Odds Ratio (OR)	95% CI	*p* value
Women	1.419	(.921–2.187)	.113	**1.678**	**(1.195–2.355)**	**.003**
Abnormal LV function	1.536	(.983–2.399)	.059	**2.126**	**(1.494–3.024)**	**.000**
PAD	1.540	(.979–2.424)	.062	1.426	(.985–2.064)	.060
**Use of LIMA graft**	**.445**	**(.289–.686)**	**.000**	**.490**	**(.345–.697)**	**.000**
**Pneumonia**	**2.782**	**(1.744–4.438)**	**.000**	**4.241**	**(2.942–6.112)**	**.000**
**Perioperative MI**	**3.901**	**(2.246–6.776)**	**.000**	**3.361**	**(2.065–5.470)**	**.000**
Previous HS	2.309	(.974–5.473)	.057	1.397	(.600–3.254)	.439
**EuroScore logistic**	**1.020**	**(1.008–1.033)**	**.002**	**1.017**	**(1.007–1.028)**	**.001**

*LV* left ventricular; *LIMA* left internal mammary artery; *MI* myocardal infarction; *PAD* peripheral artery disease

Perioperative MI (HR 3.367 for 30-day and HR 2.710 for 180-day mortality) and pneumonia (HR 2.284 for 30-day and HR 3.238 for 180-day mortality) were detected as risk factors with the highest prediction for mortality.

### Estimated survival

**Fig 3:**
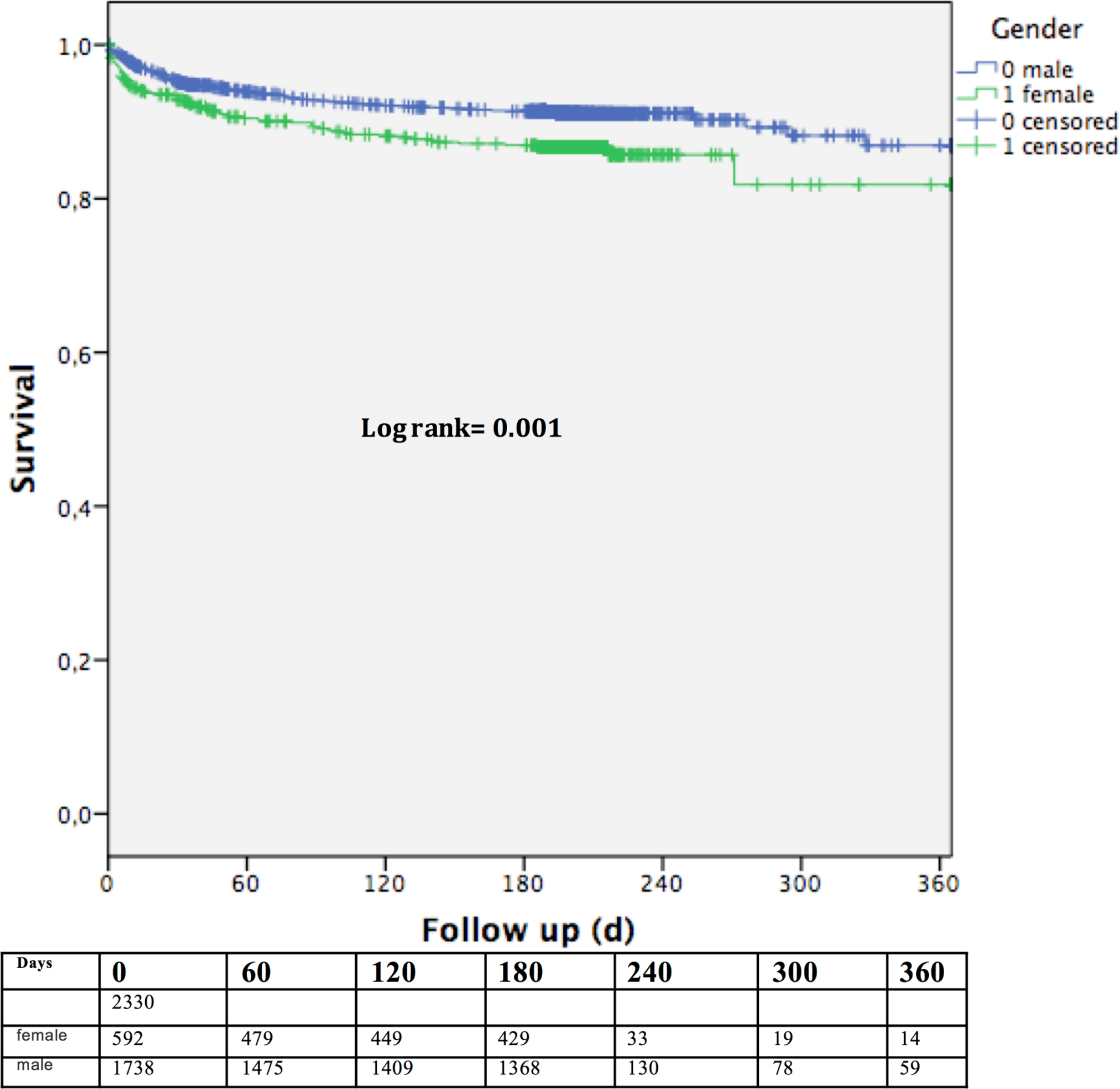
Estimates survival of septuagenarians showing a significant difference between women and men (p = 0.001).

Despite a relatively short follow-up period Kaplan-Meier log-rank test calculated significant differences in estimated survival also only among septuagenarians with an actuarial survival of 86.7 ± 1.5 in women and 91.4 ± 0.7% in men after 180 days and 81.6 ± 4.2% in women and 86.9 ± 2.2% after 360 days in men (p = 0.001) as depicted in Fig 3.

## Discussion

Aim of the study was to determine gender specific differences in outcome after CABG surgery between women and men divided into different age groups. We found significant higher observed 30-day and 180-day mortality in women compared to men only among septuagenarians. These results would underline findings of previous reports, which found female gender among other independent risk factors predicting early adverse outcome after CABG [1–5]. However, in contrast to our findings several reports observed higher mortality rates in female patients after CABG only among younger women [17, 20–22]. Various studies reported that gender-associated differences in outcome are based on differences in preoperative baseline characteristics. Physiological and clinical factors such as older age of women when admitted, smaller body size and coronary lumina and increased cardiovascular risk factors such as diabetes, arterial hypertension and hypercholesteromia contribute to gender disparity in early outcome [8, 14, 17, 23].

Surprisingly, our Cox regression analysis did not detect female gender to be an independent predictor for adverse outcome after 30-days in septuagenarians. These results confirm findings of previous studies, which reported similar results after adjustments using the propensity score, ruling out female gender as an independent predictor for early mortality [14, 15, 24]. In place of female gender other highly significant risk factors were detected. The significant risk factors remaining were logistic EuroSCORE (ES), abnormal LV function, use of LIMA graft, perioperative MI and pneumonia, which were underpinned by our results of multivariate analysis from the subgroup of septuagenarians. Using the Cox model to identify risk factors for 180-day mortality the same risk factors were detected, but furthermore, female gender became a significant risk factor (HR 1.632, CI 95% 1.212–2.200, p = 0.001). This is a surprising result, which may be caused by several factors. The delta of observed outcome increased after 180-days in disfavor of women, which may result in a higher predictive impact of female gender on adverse outcome, closing the lines to the other highly significant predictive risk factors. A not measurable factor could be the cause of death which has not been investigated in our study or the further course after discharge or transfer from our center to other smaller institutes, where we have no information of outcome details.

Still, some distinctions between women and men do exist. Women are more likely to undergo revascularization procedures later than men [3, 14, 25], which may be connected to greater fear of surgery and thus biasing data [17]. In addition, anatomical conditions such as smaller coronary vessel sizes are well-known [26, 27]. The vessel size may contribute to worse anastomosis quality especially in venous vein grafts, which could lead to the higher incidence of perioperative MI in septuagenarian women. This may emphasize the importance of LIMA graft use. Both LIMA use (HR 0.480 for 30-day and HR 0.529 for 180-day mortality) and perioperative MI (HR 3.367 for 30-day and HR 2.710 for 180-day mortality) are alongside pneumonia (HR 2.284 for 30-day and HR 3.238 for 180-day mortality) the highest predictive factors for 30-day and 180-day mortality and survival in analysis of septuagenarians and have as such also been previously reported [28]. However, the occurrence of postoperative pneumonia remains an ubiquitous risk factor after operative procedures in general. Ennker et al. pointed out the importance of arterial bypass grafts in women due to poor coronary vessel quality and capability of arterial grafts to withstand theses circumstances [23]. However, we did not find significant differences or impact on outcome of total arterial revascularization among septuagenarians. In contrast, Miśkowiec et al. recently reported that despite fewer use in women, the LIMA graft did not have a significant prediction for 30-day mortality after CABG [29]. The fact that the LIMA graft has been used significantly more among septuagenarian men may contribute to worse outcome of women in our study subgroup. However, the LIMA graft has also been used significantly more often in octogenarian men but showing no influence on differences in 30-day or 180-day mortality in this age group.

The second major prognostic factor for adverse outcome after CABG among septuagenarians is the perioperative occurrence of myocardial infarction, which has been assessed as a highly significant parameter by Cox regression analysis in this subgroup. Since it has also been found to occur significantly more in women, this factor alongside the different percentage of LIMA graft use and pneumonia may be the key reasons for the outcome differences of our cohort. Vaccarino et al. also reported that perioperative myocardial infarction is the major cause for early death in women after CABG [17]. Divided into different age groups they found the highest significant value in women of younger age. Despite decreasing differences in mortality with advancing age, a peak in outcome differences was found also among septuagenarians by Vaccarino et al. [17], which supports our results.

The baseline data analysis of our septuagenarian subgroup found men to be presenting with significantly higher prevalence of three-vessel disease, unstable angina and abnormal LV function, which one would suggest to increase mortality risk drastically. Nevertheless, our analysis merely revealed abnormal LV function among these variables as an independent predictor for 30-day and 180-day mortality in men (HR 1.566; HR 2.013). Several studies reported significant higher prevalence of comorbidities such as diabetes, renal dysfunction [16], chronic lung disease, obesity and hypertension in women [14, 25, 30]. We found only significant higher prevalence of diabetes and obesity among septua- and octogenarian women, but with no independent impact. Furthermore, other comorbidities tended to appear more frequent in men among all subgroups, especially those being statistically significant, such as abnormal LV function and peripheral artery disease. The only exception was the logistic ES, which per se calculates higher values for women due to the high impact of female gender within the score [9, 31]. The prognostic value of the ES has been discussed controversially even after further adjustment leading to ES II. ES is considered to substantially overestimate patients’ risk. Therefore other risk models have been established, e.g. the German CABG Score, which seems to be a reliable alternative to ES, with a higher predictive power [10]. The German CABG Score puts slightly less emphasis on female gender. Female gender is determined by an OR of 1.2 within the German Score and an OR of 1.4 within the ES.

Among operative variables aortic cross-clamp time was slightly longer in septuagenarian men, but showed no predictive value as in contrast being previously reported by Al-Alao et al [24]. Al-Alao et al. assumed that this might also lead to less reoperations due to bleeding complications. However, we could not confirm the findings of Al-Alao et al, as we do not observe significant differences of reoperation due to bleeding complications.

Despite a short follow-up period actuarial survival as calculated by Kaplan Meier analysis revealed also significant differences of outcome between women and men only among septuagenarians.

Our findings indicate that differences between genders in outcome after CABG vary among different age groups. Our youngest age group (sexagenarians) showed several significant differences among comorbidities, surprisingly with significant higher occurrence of possible risk factors in men, except for obesity, which was more common among women. Furthermore, besides urinary tract infections there were no significant differences in operative parameters or postoperative complications. As most of the previous reports, which described gender differences in outcome after CABG, found significant higher comorbidities among women, our baseline characteristics were more balanced between genders with a negative tendency for men. One reason for this observation may be a center related bias. Prompt indication making and alacrity of women in this age group may have led to these results. In addition, women are more aware and likely to present as early as symptoms of unstable angina emerge and men not until the state of MI [32].

In our high-risk group of octogenarians, comparable outcomes between women and men are not surprising, since high age per se is a major risk factor for mortality and may equalize differences in comorbidities between women and men. Several reports have also described this phenomenon [17, 20, 22]. Besides higher prevalence of diabetes, obesity and unstable angina no other comorbidities were significant more present in octogenarian women. However, LIMA bypass graft was significantly less used in women. Hannan et al. reported of female gender and age over 70 years being highly significant risk factors for 30-day re-admission after CABG [33]. Furthermore, the use of solitary saphenous vein graft was a significant risk factor underlining the importance of LIMA graft use. Others report of poorer patency rates of venous grafts in women compared to those in men [34]. During the postoperative course no relevant differences between genders were found in our data.

To our knowledge this is the first study to investigate possible gender related differences in outcome after CABG divided into age by decades. Gender disparity in outcome within septuagenarians was found after 180-days of follow-up, but not early postoperatively after 30-days. Not gender but co-factors such as the different use of LIMA graft and occurrence of perioperative MI influence 30-day outcome after CABG significantly. Many other underlying pathophysiological conditions may lead to our observed differences in gender. Hormonal dysfunction, menopausal changes, genetic influences and prevention bias are only few factors that have been described [35]. Reports of gender differences in outcome after elective or emergency PCI are also contradictory. There are reports of significant higher prevalence of co-morbidities in women, such as diabetes, but similar mid and long-term outcome after PCI procedures between men and women [36, 37]. As mentioned before, the emerging influence of female gender on outcome after 180-days may most likely be contributed to the fact, that the percentage of deceased women was higher after 180-days than 30-days compared to men.

### Limitations

Our available data for analysis are those commonly recorded in the medical records. We did not have information on socioeconomic variables, reproductive history, menopausal status, or behavioral and psychosocial characteristics. Lacking such data, we were unable to determine whether these factors could play a role in the mortality differences we observed. However, it can be assumed, that due to the age groups all women could be considered postmenopausal. We did not include detailed outcome information such as cause of death or information from other medical centers after patients’ discharge or transfer. Furthermore, this study is a single center study.

## Conclusions

In patients 60 years and older only septuagenarian women have an observed higher 30- and 180-day mortality risk after CABG surgery compared with men. Essential predictive risk factors for 30-day mortality are the use of the LIMA graft, perioperative MI and the prevalence of postoperative pneumonia, but not female gender. However, after 180-days of follow-up our investigation conclude that female gender becomes an independent adverse risk factor for mortality associated with CABG. Given the associated conditions in women, future efforts to maximize the use of LIMA graft and reduce the occurrence of postoperative complications such as perioperative MI and pneumonia are necessary to further improve clinical outcomes. In view of our findings, decision for surgical revascularization should not be based on gender.
